# Flat Foot in School Children

**Published:** 1914-02-07

**Authors:** 


### THE SCHOOL DOCTOR'S PROBLEMS.

### Flat Foot in School Children.

A recent report by a German school doctor draws attention to the prevalence of this defect amongst school children. We are informed that this deformity is particularly prevalent in Swiss and German schools, so much so, in fact, that it accounts for 40 per cent. of rejections amongst German recruits. In England the matter has not as yet been fully studied, largely owing to the fact that English school doctors do not pay special attention to orthopædics. Yet there is little question that it is fairly common in elementary school children, and it is therefore well to pay some attention to the point at routine examinations. The arch of the foot is usually more pronounced in normal children than it is in adults. This is due partly to the slenderness of the foot itself and to the lesser bulk of the plantar muscles. Anatomically the arch is formed by the bones of the heel and foot, and both the longitudinal and the transverse arches are supported by the ligaments, fasciæ, and muscles of the foot. The importance of the muscle tendons in maintaining the arch is well recognised, and the calf muscles play an important part in strengthening the arch. Weakening or lowering of the arch is caused by various conditions, chief of which is probably fatigue of the calf muscles and over or improper action of the plantar muscles. In a minority of cases an actual pathological condition of the bones of the foot is present.

In examining a child at the routine inspection it is important that the school doctor should be able to view his subject from the knee downwards without the intervening obscurity of boots or stockings. This is best done while the child is being weighed and measured. A glance is usually sufficient to ascertain whether or not the arch is normal; when the child stands erect with both feet placed slightly apart and toes pointing straight forward, the concavity of the arch should be such as to admit readily of the observer pushing his fingers well below it until they are impeded by the outer edge of the sole. Where this cannot be done the arch is lowered. The extent and nature of this lowering must then be ascertained. It is best to proceed carefully and not to depend on rough and ready eye estimations. The provision of a few sheets of paper painted with iron perchloride solution and a small bottle of a solution of tannic acid in water is all that is required in the way of apparatus. The child's foot is lightly brushed over with the tannic-acid solution, and he is told to tread firmly and evenly on the iron-painted paper. The result, if this simple operation is carefully done, is an indelible impression of the foot on the paper, which can be filed for future reference with the child's record. The normal impression gives the heel and toes boldly outlined, with merely a strip of the outside edge of the foot; when there is any lowering of the arch more of the sole is traced on the paper, until, in extreme cases of flattening, the whole outline of the sole is printed on the paper.

Tested by this method, it was found that among 200 boys no fewer than seventy showed a lowering of the arch in one or both feet. In a child of ten such a lowering is a serious matter, which should be remedied while there is yet time. When the flattening is unilateral it leads to a compensatory lateral curve of the back, which is very often noted on the record card without its cause being ascertained. We have seen many such cases of lateral curvature treated by exercises and massage without the primary defect responsible for it being corrected first. This is an obviously mistaken therapy, which cannot prove successful. The proper method to deal with such cases is to correct the deformity of the foot, and to treat the lateral curvature as a secondary complication.

The treatment of flat foot in school children must depend on the extent and degree of deformity present. Where the flattening is relatively small, it is probable that a faulty carriage of body and a faulty position of the feet are responsible. Injudicious drilling exercises may occasion this. The silly habit of ordering children to turn their toes out when they walk, and thus produce an eversion of the foot, is undoubtedly one of the most frequent causes of flat foot in children. Another common cause is the wearing of heavy boots with flabby uppers and doubly-clumped soles; these tire the muscles of the foot and cause it to assume a position of ease, which is a slight eversion. Long marches when the child is tired are another cause. Flat foot in scouts and in full-bodied, bulky children are perhaps more often due to this than to any other cause. In girls, bad positions taken up in the dancing lesson account for some cases, but with them the most common cause is probably anæmia and fatigue causing weak calf muscles.

Inquiry should be made as to possible causes of the nature just alluded to in every case. In a few it is as well to inquire into recent illnesses of the child; exertion after an illness like scarlet fever, diphtheria, or mumps, when the general muscle tone is lax, may cause a temporary flattening of the arch, which, in course of time, unless treated, becomes permanent. In a few cases no cause can be discovered or traced. Where a cause has been noted the child should be told of it, and warned about the ill effects resulting from a persistence in the attitude or habit. A little lecture on the foot is not out of place in dealing with such cases; the child is easily interested in the matter, and can as easily be turned into a helpful assistant in the treatment. Then something must be done to correct the deformity. Exercises are usually recommended, and even the text-books still give elaborate rules with regard to these exercises.

As a general rule it may be stated that while proper exercises, tending to correct the eversion, are of use in slight cases, they are not the slightest good in cases where the flattening is of any marked extent. Then the arch must be supported by artificial means. By all means strengthen the muscles of the calf by massage and exercise, but a regular course of foot exercise usually tends to aggravate the flattening and does little to restore the integrity of the foot. The nature of the artificial support is of some consequence. The best is undoubtedly a celluloid or cork inset, analogous to the Whitman's brace, modelled from a plaster cast of the child's foot corrected at the time of taking the mould. Such a celluloid inset can easily be made by any instrument maker; it is light, strong, and with sufficient spring, and can be slipped into any boot or shoe. Properly made it can be worn safely for from six months to a year; then it has to be renewed. Its cost, in comparison with the leather and brass braces usually sold ready-made by instrument makers, is small, and it is a far more reliable and efficient instrument than these ready-made articles. The wearing of such an inset eases the foot and gives the necessary support to the arch. In slight cases it may effect a cure; in more severe cases it prevents the deformity from increasing.

